# Core–Shell Prussian Blue Analogs with Compositional Heterogeneity and Open Cages for Oxygen Evolution Reaction

**DOI:** 10.1002/advs.201801901

**Published:** 2019-02-08

**Authors:** Wuxiang Zhang, Hao Song, Yan Cheng, Chao Liu, Chaohai Wang, Muhammad Abdul Nasir Khan, Hao Zhang, Jizi Liu, Chengzhong Yu, Lianjun Wang, Jiansheng Li

**Affiliations:** ^1^ Jiangsu Key Laboratory of Chemical Pollution Control and Resources Reuse School of Environmental and Biological Engineering Nanjing University of Science and Technology Nanjing 210094 P. R. China; ^2^ Australian Institute for Bioengineering and Nanotechnology The University of Queensland Brisbane QLD 4072 Australia; ^3^ Department of Electronic Engineering School of Information Science Technology East China Normal University Shanghai 200241 P. R. China; ^4^ School of Chemistry and Molecular Engineering East China Normal University Shanghai 200241 P. R. China; ^5^ Herbert Gleiter Institute of Nanoscience Nanjing University of Science and Technology Nanjing 210094 P. R. China

**Keywords:** bimetallic Prussian blue, core–shell nanocages, oxygen evolution reaction, reduction‐cation exchange

## Abstract

Here, a reduction‐cation exchange (RCE) strategy is proposed for synthesizing Fe–Co based bimetallic Prussian blue analogs (PBAs) with heterogeneous composition distribution and open cage nanocage architecture. Specially, bivalent cobalt is introduced into a potassium ferricyanide solution containing hydrochloric acid and polyvinyl pyrrolidone. The uniform PBAs with opened cages are formed tardily after hydrothermal reaction. Time‐dependent evolution characterization on composition elucidating the RCE mechanism is based on the sequential reduction of ferric iron and cation exchange reaction between divalent iron and cobalt. The PBA structures are confirmed by electron tomography technology, and the heterogeneous element distribution is verified by energy‐dispersive X‐ray spectroscopy elemental analysis, leading to the formation of core–shell PBAs with compositional heterogeneity (Fe rich shell and Co rich core) and open cage architecture. When the PBA catalysts are used to boost the oxygen evolution reaction (OER), superior OER activity and long‐term stability (low overpotential of 271 mV at 10 mA cm^−2^ and ≈5.3% potential increase for 24 h) are achieved, which is attributed to the unique compositional and structural properties as well as high special surface areas (576.2 m^2^ g^−1^). The strategies offer insights for developing PBAs with compositional and structural multiplicity, which encourages more practical catalytic applications.

## 1 Introduction

The development of hierarchical nanomaterials with controllable composition and morphology has provided new opportunities to tune physical/chemical properties, making them promising candidates for both fundamental studies and practical applications.^1^, ^2^, ^3^, ^4^ Recently, numerous types of nanomaterials with complex nanostructures (NCSs), such as core–shell,^5^, ^6^ yolk–shell,^7^ multishell, and open‐cage/frame architectures,^8^, ^9^ have attracted considerable interests because of their intrinsic properties and potential applications in diverse fields.^10^, ^11^, ^12^, ^13^, ^14^, ^15^, ^16^, ^17^, ^18^ Prussian blue analogs (PBAs), a subclass of the metal–organic frameworks, are a new platform for the synthesis of NCSs.^19^, ^20^ PBAs are constructed by octahedral M_1_[M_2_(CN)_6_], where M_1_/M_2_ represent transition metal cations (e.g., Fe, Co, Ni, Mn, Zn, etc.).^21^, ^22^ To date, several strategies have been developed for the preparation of bimetallic PBAs with adjustable architectures, including soft/hard templating,^23^, ^24^ chemical etching,^25^, ^26^ element replacement,^27^, ^28^ and self‐templated epitaxial growth,^29^, ^30^ leading to PBAs with a variety of compositions and morphologies (summarized in Table S1 in the Supporting Information). However, previous reports focus on the synthesis and structure–performance relationship of PBAs with a homogenous or single‐phase composition. There are rare reports on PBAs with compositional heterogeneity and their applications.

In addition to the nanostructure complexity, the compositional heterogeneity of nanomaterials has significant impact on their properties. For instance, Jung et al.^31^ reported that the edge‐ and corner‐embedded bimetallic nanocubes achieved outstanding oxygen reduction reaction and oxygen evolution reaction (OER). Burke et al.^32^ demonstrated that Fe cations at edges or defects of cobalt (oxy)hydroxide are responsible for the enhanced OER activity. Stevens et al.^33^ reported that increasing the number of Fe edge or defect sites on Ni oxyhydroxide improved the oxygen electrocatalysis activity. It is hypothesized that PBAs with both complex nanostructures and compositional heterogeneity could have improved performance. Moreover, in most reports two‐step or multistep processes are needed to prepare PBAs with specific structures.^25^, ^26^, ^34^, ^35^, ^36^ Developing a facile approach to synthesize PBAs with both nanostructure complexity and compositional heterogeneity is still imperative and highly desired.

**Figure 1:**
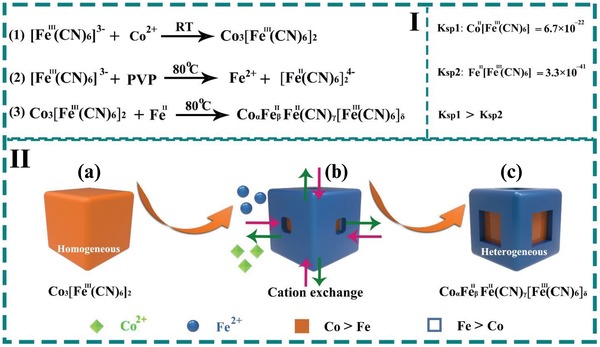
Reaction scheme for the preparation of core–shell PBAs.

Herein, we report a one‐pot synthesis of Fe–Co PBAs with both a heterogeneous composition distribution and a core–shell architecture with open cages. The fabrication procedure of Fe–Co PBAs is depicted in **Figure** **1**. First, bivalent cobalt and potassium ferricyanide solution were mixed in an polyvinylpyrrolidone (PVP) and acid solution, the Co_3_[Fe^III^(CN)_6_]_2_ nanocubes were formed rapidly at room temperature (Figure 1I, reaction 1, Figure 1II‐a ). Next, the same reaction system was subjected to hydrothermal reaction at 80 °C for different time (*t*, *t* = 1 min, 1, 4, 12, and 24 h) to give the final products (named as PBA 1–5, respectively). The partial reduction of [Fe(CN)_6_]^3−^ by PVP as the reducing reagent produced Fe^II^ species (Figure 1I, Reaction (2)). Because the solubility precipitation constant (Ksp) of Fe^II^_3_[Fe^III^(CN)_6_]_2_ (Ksp = 3.3 × 10^−41^) is smaller than that of Co_3_[Fe^III^(CN)_6_]_2_ (Ksp = 6.7 × 10^−22^, inset of Figure 1I),^27^ Fe^II^ selectively etched the face‐center positions due to the selective accumulation of PVP at edge/corner sites,^36^, ^37^, ^38^ leading to the formation of PBA‐5 (Figure 1I, Reaction (3)) with both complex nanostructure (core–shell cubes with open cages) and compositional heterogeneity (Fe‐rich shell and Co‐rich core, Figure 1II, Figure 1II‐b,c). We demonstrate that both the structure and composition can be adjusted by hydrothermal treatment. The obtained PBA‐5 catalysts with a high specific surface area of 576.2 m^2^ g^−1^ exhibited excellent OER activity and long‐term stability (low overpotential of 271 mV at 10 mA cm^−2^ and ≈5.3% potential increase for 24 h).

## 2 Results and Discussion

**Figure 2:**
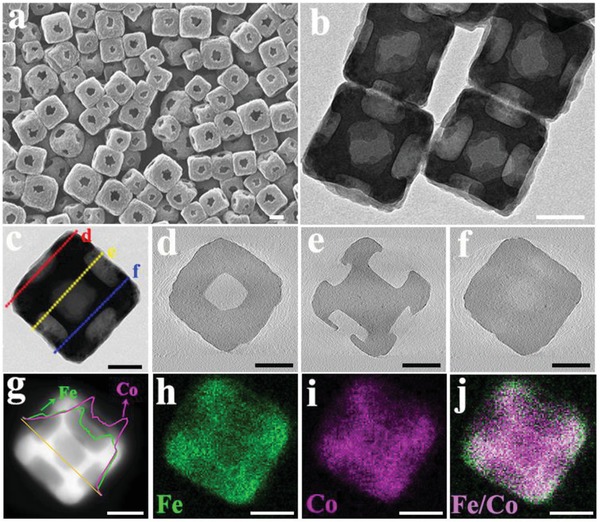
a) FESEM, b,c) TEM and ET slides in d) top, e) central, and f) core–shell connection section, respectively. g) HAADF images with EDX line‐scans obtained along the middle line scanning of PBA‐5 and elemental maps of h–j) Fe, Co, and Fe–Co merged images (All scale bar at 100 nm).

The structures of Fe–Co PBA‐5 were characterized by field‐emission scanning electron microscopy (FESEM) and transmission electron microscopy (TEM). FESEM image (**Figure** **2**a) shows that PBA‐5 possesses a well‐defined cubic shape with an open hole in the face‐center position in large domains. The particle diameter was measured to be ≈230 nm. From the TEM image of PBA‐5 (Figure 2b), the existence of cavity with size of ≈64 nm on the surface is clearly observed, in agreement with the scanning electron microscopy (SEM) result. These cavities lead to low mass‐thickness contrast in the center of the nanocube under TEM analysis, appearing as “hollow” structures. However, the flattened 2D TEM image can hardly reveal the real architecture of this nanoparticle,^39^ especially for these nanocubes aligned at a particular angle under the electron beam.

To further investigate the complex interior structures of PBA‐5, nanoparticles were characterized under TEM along varied tilting angles and then processed for electron tomography (ET) analysis (Figure S1 and Video S1, Supporting Information). The nanocube exhibits a “hollow” cavity in the center of the particle as observed at the angle of 0°. When the specimen is rotated under the angle of ± 30°, the nanoparticle shows an intricate cage‐like nanostructure. By further tilting the nanocube to angles of ± 60°, a solid core can be clearly identified linked to the outer shell of the nanoparticle. Tomographic analysis of this nanocube further reveals the complexity of its interior nanostructure. Tomograms sliced at position d,–f as indicated in Figure 2c are shown in Figure 2d–f, respectively. Slicing through the top of the nanocube, the surface cavity with an average window size of ≈64 nm and depth of ≈50 nm can be identified in the ET slice (Figure 2d), in accordance with the observations under FESEM and TEM (Figure 2a,b). When the nanocube is sliced through the center, the tomogram (Figure 2e) reveals a solid core with a diameter about 120 nm, as well as large cavities on each surface of the nanocube. Interestingly, when the nanoparticle is sliced along the adjacent between the solid core and cage‐like shell at selected position f, the ET slice appears as an intact solid cube. The real structure of PBA‐5 can be reconstructed, showing a solid‐core/cubic‐shell structure with cavities on each facet (ET analysis and 3D reconstruction shown in Video S2 in the Supporting Information).

To study the chemical composition of PBA‐5, the high‐angle annular dark‐field (HAADF) image and corresponding element analysis spectra are presented in Figure 2g. Energy‐dispersive X‐ray spectroscopy (EDX) line‐scanning spectra show a higher signal intensity of Fe than that of Co in the outer region of cubic shell, indicating a Fe‐rich shell of PBA‐5. By contrast, a Co rich core is found inside the outer shell of PBA‐5. The element mapping images of Fe, Co, and their overlay (Figure 2h–j) further confirm that higher content of Fe is distributed on the outer edge of PBA‐5. To further characterize the compositional heterogeneity of PBA‐5, the spatial distribution of Fe and Co elements was also analyzed by EDX tomography. The 3D elemental distribution was visualized by subsequent reconstruction and shown as Figure S2 and Video S3 in the Supporting Information, where the red and green colors represent the iron and cobalt elements, respectively. From the EDX tomography results, the PBA‐5 nanoparticle has a core rich in Co, while shell (especially the edge and corner sites) rich in Fe, consistent with the results of EDX analysis of line‐scans and mappings (Figure 2g–j).

To understand the structure and formation of PBA‐5, systematic investigations have been carried out. First, a synthesis under room temperature without other changes compared to that of PBA‐5 was conducted, resulting in the generation of Co–Fe bimetallic PBA room temperature (denoted as PBA‐RT). The SEM and TEM images (Figure S3a,b, Supporting Information) display a solid‐cube structure of PBA‐RT without open cage on the outer shell. The X‐ray diffraction (XRD) pattern of PBA‐RT possesses characteristic peaks at 17.15°, 24.3°, 34.7°, and 39.0°, which can be indexed the (100), (110), (200), and (210) diffractions of Co–Fe bimetallic PBA, respectively. This result is in consistent with previous literature reports.^40^, ^41^, ^42^ Moreover, differ from the compositional heterogeneity of PBA‐5, the EDX line‐scanning spectra and EDX points analysis show the homogeneous Fe and Co distribution in the whole framework of PBA‐RT, indicating the composition homogeneity (shown in Figure S3c–f in the Supporting Information). Therefore, under the RT condition, Fe^3+^ cannot be reduced to Fe^2+^ due to the relatively mild reaction environment. PBA‐RT with a pure Co_3_[Fe^III^(CN)_6_]_2_ phase is formed (Figure 1II‐a) as the starting particle in the following cation‐exchange and etching process.

Next, the products at different hydrothermal treatment time (*t*, *t* = 1 min, 1, 4, 12, and 24 h, Figure S4, Supporting Information) were monitored. After 1 min, a solid cubic shape of PBA‐1 with a uniform size of ≈230 nm was obtained (Figure S4a, Supporting Information). As the hydrothermal reaction time was increased to 1 h, a small hole about 35 nm diameter was generated on the central face of PBA‐1 (Figure S4b, Supporting Information). Increasing *t* to 4–24 h leads to gradually enlarged central cavities (Figure S4c–e, Supporting Information), finally forming PBA‐5. Moreover, even the hydrothermal synthetic process prolonged to 15 d (PBA‐6), negligible changes of the crystal structural and composition were observed (Figure S5, Supporting Information), indicating that the reaction equilibrium was achieved at 24 h. The corresponding structural features of PBA‐*t* are summarized in Table S2 in the Supporting Information. The particle sizes of PBA‐*t* were also quantitatively measured (Figure S6, Supporting Information). The average particle sizes are about 230 nm for all PBA‐*t*, indicating that only the face‐center was etched during the reaction.

**Figure 3:**
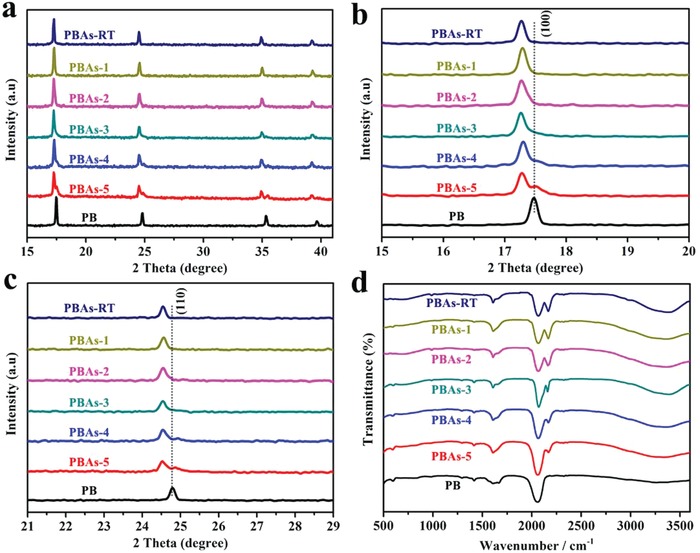
a) XRD patterns, XRD enlarged patterns of b) 100 faces, c) 110 faces, and d) FTIR spectra of PBA‐1, PBA‐2, PBA‐3, PBA‐4, and PBA‐5. The initial product PBA‐RT after 24 h reaction at room temperature without hydrothermal treatment and conventional PB as a control were also included.

The XRD patterns of PBA‐*t* were shown in **Figure** **3**a. Compared to pure Co_3_[Fe^III^(CN)_6_]_2_, new peaks appeared as shoulders with their intensity increased with the hydrothermal reaction time, which can be indexed to Fe_3_[Fe^III^(CN)_6_]_2_ as compared to the XRD pattern of pure Fe_3_[Fe^III^(CN)_6_]_2_. The enlarged figure of (100) and (110) diffractions of PBA‐*t* is shown in Figure 3b,c, where the shoulders are clearly seen. The lattice distance (*d*) of Fe_3_[Fe^III^(CN)_6_]_2_ is smaller than that of Co_3_[Fe^III^(CN)_6_]_2_, e.g., *d* (100) decreased from 5.16 to 5.08 Å, and *d* (110) decreased from 3.64 to 3.59 Å, which is explained by the smaller size of Fe^2+^ compared to Co^2+^. For PBA‐5, the intensity of (100) and (110) diffractions assigned to Fe_3_[Fe^III^(CN)_6_]_2_ is much lower than that of Co_3_[Fe^III^(CN)_6_]_2_, suggesting Fe_3_[Fe^III^(CN)_6_]_2_ is still a minor phase, while Co_3_[Fe^III^(CN)_6_]_2_ is dominant in the product.

Fourier transform infrared spectroscopy (FTIR) spectrums of PBA‐*t* are displayed in Figure 3d. For Co_3_[Fe^III^(CN)_6_]_2_ (PBA‐RT), the characteristic peaks at 2081 and 2160 cm^−1^ are attributed to the cyan bond stretching mode associated with pure PB^41^, ^42^ and Co^II^‐CN‐Fe^III^,^30^ respectively. Compared to pure Co_3_[Fe^III^(CN)_6_]_2_, the peak intensity at 2160 cm^−1^ for PBA‐*t* decreased, while that at 2081 cm^−1^ increased with the hydrothermal reaction time, indicating the gradual transformation from the initial Co_3_[Fe^III^(CN)_6_]_2_ phase to a Fe_3_[Fe^III^(CN)_6_]_2_ and Co_3_[Fe^III^(CN)_6_]_2_ mixed phase in the products. In addition, the color of the PBA‐*t* changed from brownish (PBA‐1) to light red (PBA‐2) and finally to blue (PBA‐5), further suggesting the continuous replacing of Co^2+^ with Fe^2+^ (Figure S7, Supporting Information). N_2_ adsorption–desorption isotherms of PBA‐1–5 together with their pore volume are shown in Figure S8 in the Supporting Information. The Brunauer–Emmett–Teller (BET) surface areas and pore volume of PBA‐1–4 are 454.7 m^2^ g^−1^ and 0.33 cm^3^ g^−1^, 461.9 m^2^ g^−1^ and 0.34 cm^3^ g^−1^, 468.6 m^2^ g^−1^ and 0.37 cm^3^ g^−1^, 484.7 m^2^ g^−1^ and 0.39 cm^3^ g^−1^, respectively (Table S3, Supporting Information). The PBA‐5 presented a high surface area of 576.2 m^2^ g^−1^, a large pore volume of 0.42 cm^3^ g^−1^, along with both micropores (0.6–2 nm) and mesopores (2–4 nm). The results indicate that the specific surface area and porous structure have an obvious improvement in the hydrothermal process.

**Figure 4:**
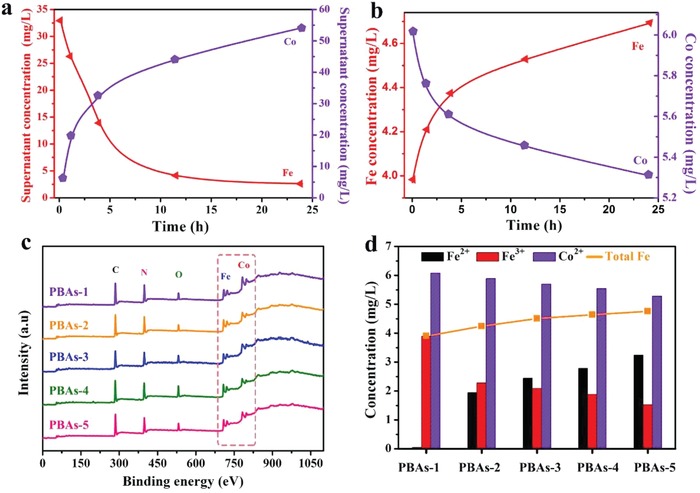
a) Concentration of total Fe and Co in the supernatant solutions. b) Total Fe, Co concentration of PBA nanoparticle after nitrolysis. c) Full XPS spectra of PBA‐1–5 nanoparticles. d) The concentration of Fe^2+^ and Fe^3+^, Co^2+^, respectively.

The overall composition change during the synthesis was monitored by measuring the contents of Fe and Co elements in PBA‐*t* samples and the corresponding supernatants where the powders were prepared by inductively coupled plasma optical emission spectrometer (ICP‐OES). The dramatically lower concentration of Co ions (C_Co_) in the supernatants (5.4 mg L^−1^) at 1 min (**Figure** **4**a) compared to the initial C_Co_ (1750 mg L^−1^) is due to the consumption of Co^2+^ for generating Co_3_[Fe^III^(CN)_6_]_2_. With increasing reaction time, the C_Co_ of in the supernatants of PBA‐2–5 increased from 19.7, 32.8, 46.3 to 53.4 mg L^−1^, respectively. On the contrary, C_Fe_ in the supernatants of PBA‐1–5 decreased from 33.2, 26.2, 14.6, 3.08 to 2.64 mg L^−1^, respectively, consistent with the Co^2+^ replacement mechanism with Fe^2+^. For PBA‐1–5 powder samples (10 mg L^−1^ total) (Figure 4b), the changes in C_Co_ and C_Fe_ showed an opposite tread with gradually increased C_Fe_ (from 4.0 to 4.7 mg L^−1^) and decreased C_Co_ (from 6.0 to 5.3 mg L^−1^) with reaction time. The EDX measured on powder samples is also consistent with the results from ICP‐OES data (Figure S9, Supporting Information). The presence of K element was also detected by EDX analysis. As shown in Figure S9 in the Supporting Information, a relatively low content of K (≈1.5 wt%) was observed in five PBA samples, indicating that K^+^ can easily migrate into the framework of PBAs, in accordance with a previous report.^13^

X‐ray photoelectron spectroscopy (XPS) survey was also employed to investigate the valence states in PBA‐*t*. The survey scan spectrum manifests that all the PBA‐*t* are composed of Co, Fe, C, N, and O elements (Figure 4c). From the high‐resolution XPS spectrum in Figure S10 in the Supporting Information, it can be seen that the Co 2p peaks were found at 782.0 eV (Co 2p_3/2)_, 786.4 eV (Co 2p_3/2_), 797.6 eV (Co 2p_1/2_), and 803.7 eV (Co 2p_1/2_), indicating the existence of Co^2+^ in the framework.^43^, ^44^, ^45^ High‐resolution Fe 2p spectrum in PBA‐2–5 gives four peaks at 708.4 eV (Fe^2+^ 2p_3/2_), 709.8 eV (Fe^3+^ 2p_3/2_), 721.3 eV (Fe^2+^ 2p_1/2_), and 724.3 eV (Fe^3+^ 2p_1/2_), corresponding to Fe^2+^ and Fe^3+^, respectively, while only Fe^3+^ was detected in PBA‐1. According to the XPS and ICP‐OES results of PBAs, the statistics graphs of C_Co_, C_Fe3+_ , and C_Fe2+_ were calculated in Figure 4d, it shows that the C_Fe2+_ gradually increased along with the decrease of C_Co_ and C_Fe3+_. These results are in agreement with the mechanisms shown in the reactions 2 and 3 in Figure 1I. Their calculating formulas were expressed by Co*_α_*Fe^II^ *_β_*[Fe^II^(CN)_6_]*_γ_*[Fe^III^(CN)_6_]*_δ_* equation, where α, β, γ, and δ were calculated according the results of ICP‐OES, XPS, and charge balanced (shown in Table S4 in the Supporting Information).^46^ For example, PBA‐5 has an calculating formula of Co_2.59_Fe^II^_0.61_[Fe^II^(CN)_6_]_1.03_[Fe^III^(CN)_6_]_0.76_. In addition, the Fe^2+^/Fe^3+^ ratio of PBA‐5 was also verified by Mössbauer spectroscopy. As shown in Figure S11 in the Supporting Information, the obtained spectrum could be fitted into two fitting curves by Lorentzian lines, which were associated with the low‐spin iron(II) and high‐spin iron(III) spectral components.^47^ The resulting spectral fitting parameters are summarized in Table S5 in the Supporting Information. The fitting amounts of the Fe^2+^ (68%) and Fe^3+^ (32%) were found, which was consistent with the results of XPS (Figure 4d). Interestingly, the reduction‐cation exchange (RCE) approach can be used to generate solid bimetallic Fe–Mn PBAs with adjustable Fe/Mn ratios (Figure S12, Supporting Information). This is because Mn PBAs cannot be formed prior to the hydrothermal treatment to induce the RCE process due to it higher solubility product constant (Ksp = 1.9 × 10^−3^, see detailed discussions in Figure S12 in the Supporting Information and captions).^27^ It is suggested that the preformation of Co_3_[Fe^III^(CN)_6_]_2_ nanocubes prior to the RCE process is critical to the formation of the core–shell structures of Fe–Co PBAs.

**Figure 5:**
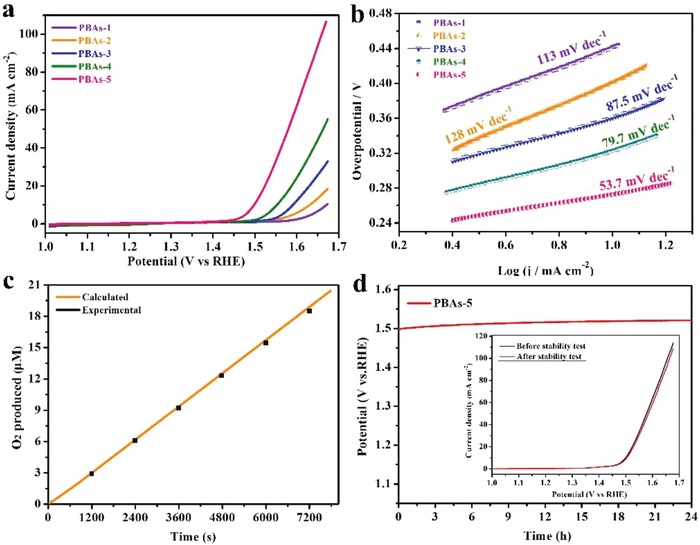
a) LSV curves and b) Tafel slopes of PBA‐1–5 electrodes, c) actual number of O_2_ generated versus calculated values by PBA‐5 catalysts at constant current of 1 mA electrolysis. d) Chronopotentiometry curve of PBA‐5 electrode at a current density of 10 mA cm^−2^. Inset: compared with the LSV curves of PBA‐5 before and after 1000 cycles.

The electrocatalytic OER activities of PBAs samples were investigated in 1 m KOH electrolyte using a standard three‐electrode system. Previously, the electrodes were activated by cyclic voltammetry at a sweep rate of 10 mV s^−1^, which make sure that the measured electric current is attributed to OER in the polarization curves rather than catalysts themselves. The linear sweep voltammetry (LSV) curve are shown in **Figure** **5**a, as expected, PBA‐5 exhibits a low overpotential of 271 mV at 10 mA cm^−2^, which is smaller than that of PBA‐1 (440 mV), PBA‐2 (402 mV), PBA‐3 (364 mV), and PBA‐4 (321 mV). For comparison, similar measurement for the single Fe, Co‐based PB sample, PBA‐6 and commercial RuO_2_ was also performed in Figure S13a in the Supporting Information. The negligible current density toward OER between single Fe‐ and Co‐based PB, indicated that a single Fe‐ and Co‐based PB is unavailable for OER catalysis. The polarization curve of PBA‐6 is well in accordance with that of PBA‐5, further verified the reaction equilibrium was achieved at 24 h. The commercial RuO_2_ catalyst requires an overpotential of 288 mV to reach a current density of 10 mA cm^−2^, revealing a favorable OER activity of PBA‐5 catalyst. Further insights into the OER kinetics were analyzed through Tafel plots in Figure 5b, based on the Tafel equation η = b log (j), where η is the overpotential, b is the Tafel slope, and j is the current density. The Tafel slope of the PBA‐5 (53.7 mV dec^−1^) was obviously lower than that of the PBA‐1 (113 mV dec^−1^), PBA‐2 (128 mV dec^−1^), PBA‐3 (87.5 mV dec^−1^), PBA‐4 (79.7 mV dec^−1^), and less than the commercial RuO_2_ (76.3 mV dec^−1^) catalysts (shown in Figure 5b and Figure S13b in the Supporting Information). A detailed comparison of OER activities of representative bimetallic‐based catalysts is summarized in Table S6 in the Supporting Information, suggesting that PBA‐5 prepared in our study exhibit a comparable OER performance to benchmark materials.

To reveal the charge‐transfer resistance (*R*_CT_) kinetics on PBA catalysts, electrochemical impedance spectroscopy was analyzed in high‐frequency semicircle region (Figure S14 and Table S7, Supporting Information), the PBA‐5 shows the smallest semicircle values in the Nyquist plots (2.3 Ω), suggesting the PBA‐5 have relatively lower charge transfer resistance and considerable electron‐transfer rate and thus the enhanced the OER activity. Faradaic efficiency experiment of PBA‐5 electrocatalyst catalysts was further conducted in Figure 5c. The amount of O_2_ evolved during constant current electrolysis is matched well with the calculated charge values, indicating a near 100% Faradaic efficiency is obtained. The remarkable OER actives on PBA‐5 should be ascribed to the combination effect between the composition and structure of PBA‐5. Compositionally, a proper ratio of Fe/Co hybrid material with synergistic effects can significantly improve electrocatalytic activity.^48^, ^49^, ^50^ Structurally, the open cage structure, large amount of pore volume with high special surface areas (576.2 m^2^ g^−1^), and low charge‐transfer resistance (2.3 Ω) could speed up the electron transfer. All these advantages above could boost the excellent OER performance.

Besides the high catalytic activity, stability study is vital basis to evaluate the practical application of the catalyst. The PBA‐5 exhibits the prominent stability with a constant overpotential of 271 mV, only a slight decrease (about 5.3%) was given after 24 h, suggesting the robustness stability of the catalyst (Figure 5d). In addition, The PBA‐5 held the LSV polarization curve barely shifts than the initial one with negligible loss in overpotential and current density (inset of Figure 5d). The morphologies of the PBA‐5 catalysts after being used in 24 h chronopotentiometry assessment were characterized by SEM and TEM (Figure S15a,b, Supporting Information), the well‐maintained open cage structure further indicates its stability. No apparent crystal structure and compositional change were observed as evidenced by XRD (Figure S15c, Supporting Information) and XPS results (Figure S15d,e, Supporting Information), suggesting that the PBA‐5 catalysts are stable and durable OER catalysts. However, high‐resolution O1s peak in the XPS spectrum showed the appearance a weak ‐OH shoulder at 530.8 eV (Figure S15f, Supporting Information),^51^, ^52^ indicating the partial transformation into hydroxides occurred at the surface of PBA‐5 after the electrochemical process.^53^ Therefore, the PBA‐5 catalysts with compositional heterogeneity and open cages are available for efficient and durable OER performance.

## 3 Conclusion

In summary, the core–shell Prussian blue analogs with compositional heterogeneity and open cages for oxygen evolution reaction were successfully fabricated and the mechanism was studied in detail by time‐dependent evolution process. The obtained PBA‐5 materials exhibit an open cage, Fe rich in shell, and Co rich in the solid core with a high cavity volume, high special surface areas (576.2 m^2^ g^−1^). Additionally, a RCE mechanism was proposed. Due to the structural and compositional advantages, the heterogeneous PBA‐5 show enhanced electrocatalytic activity for OER. The present study may inspire further capability on constructing complex structured PBA varieties and may further extend their applications to other energy‐related applications.

## 4 Experimental Section

*Materials*: Potassium hexacyanoferrate (III) [K_3_Fe(CN)_6_], hydrochloric acid, and PVP (K30, *M*_W_ ≈ 40 000) were purchased from Sinopharm Chemical Reagent Co., Ltd. Cobalt nitrate was purchased from Sigma‐Aldrich. All chemicals used in the experiments were of analytical grade without further purification. The deionized water was used for all the experiments.

*Material Preparation—Synthesis of Fe–Co PBAs*: Materials synthesized using potassium hexacyanoferrate (III) as a frame. In brief, PVP was dissolved in HCl solution (0.1 m, 40 mL), then, divided evenly into two equal parts. K_3_Fe(CN)_6_·3H_2_O (65 mg) was added into the above solution (20 mL) under magnetic stirring for 10 min (solution A), Co(NO_3_)_2_·6H_2_O 70 mg was dissolved into the above solution (20 mL) for 10 min magnetic stirring to obtain a clear solution (solution B). Then, mixing into the two solutions to beaker and the color of the final solution became brownish red. After that the final solution was stored into the electric oven at 80 °C for 24 h. During the synthesis process, the color of the solution changed from brownish to blue. Finally, the resultant products were centrifuged, washed with deionized water and ethanol for several times, and dried for 24 h in a vacuum oven at 50 °C. The as‐prepared PBA‐*t* using different stages (1 min, 1 h, 4 h, 12 h, 48 h, and 15 d) were denoted as PBA‐1, PBA‐2, PBA‐3, PBA‐4, PBA‐5, and PBA‐6, respectively.

*Material Preparation—Synthesis of PBA‐RT*: PVP was dissolved in HCl solution (0.1 m, 40 mL) under magnetic stirring, then, divided evenly into two equal parts. K_3_Fe(CN)_6_·3H_2_O (65 mg) was added into the above solution (20 mL) under magnetic stirring for 10 min (solution A), Co(NO_3_)_2_·6H_2_O 70 mg was dissolved into the above solution (20 mL) for 10 min magnetic stirring to obtain a clear solution (solution B), then, mixing into the two solutions and aged at RT for 24 h.

*Material Preparation—Synthesis of Prussian Blue (PB)*: 2.0 g of PVP was dispersed in HCl solution (0.1 m, 20 mL) under stirring then K_3_Fe(CN)_6_·3H_2_O (0.065 g) was added, and the solution kept under stirring for more 20 min. Following, the final solution was placed into an electric oven and heated at 80 °C for 24 h. The obtained blue PB product was centrifuged and washed several times with deionized water and ethanol, and finally dried in a vacuum oven at 50 °C for 24 h.

*Material Preparation—Synthesis of Single Co Prussian Blue (Co–Co PB)*: 2.0 g of PVP was dissolved in HCl solution (0.1 m, 20 mL) under stirring, then 70 mg potassium hexacyanocobaltate(III) was added, cobaltous nitrate of 50 mg was dissolved into the above solution and kept under stirring for more 20 min. Following, the solution was placed into an electric oven and heated at 80 °C for 24 h. The obtained Co–Co PB product was centrifuged and washed several times with ethanol, and finally dried in a vacuum oven at 50 °C for 24 h.

*Material Preparation—Synthesis of Fe–Mn PBAs*: PVP was dissolved in HCl solution (0.1 m, 40 mL). Then, the mixture solution was divided evenly into two parts. K_3_Fe(CN)_6_·3H_2_O (65 mg) was added into one part of the solution (20 mL) under stirring for 10 min (solution A), Mn(NO_3_)_2_ 70 mg was dissolved into another part (20 mL) also under stirring to obtain a clear solution (solution B). Afterward, solutions A and B were mixed together under stirring at room temperature. The final solution was transferred into an oven and hydrothermally treated at 80 °C under static conditions. Fe–Mn‐PBA‐1 and Fe–Mn‐PBA‐2 were obtained when the hydrothermal reaction time was 5 min and 12 h, respectively. The products were centrifuged and washed three times with ethanol, and finally dried in a vacuum oven at 50 °C for 24 h.

*Characterization*: The morphologies of all products were characterized by using a SEM (FEI Quanta 250F system) operating at 20 kV. TEM was performed on a FEI Tecnai G20 electron microscope (FEI Company, USA) operating at 300 kV. ET specimens were carried out by tilting the specimen inside the microscope around core axis from +60° to −60° under the electron beam. 3D reconstructions and alignment of PBAs were implemented on IMOD software. EDX tomography was performed on Grand ARM300F with an operating voltage of 80 kV. EDX mapping images were collected by tilting the specimen from −51.5° to +60.5° with a tilting interval of 8° per tilt. The 3D reconstruction was realized by processing the EDX data via software of Visualizer‐Kai. The time for capturing the elemental signal at each tilt was 5 min. The large tilt interval of 8° was chosen due to the instability of PBAs under electron beams even at a low operating voltage of 80 kV. X‐ray powder diffraction (XRD) patterns of all sample were recorded on the Bruker D8 Advance at 40 kV and 40 mA (Bruker AXS, Germany), X‐ray diffractometer at a scan rate of 0.05 dec s^−1^ using Cu Kα radiation (λ = 1.5418 Å). FTIR patterns of all samples were recorded on the Bruker (Bruker HYPERION, Germany). Nitrogen adsorption/desorption isotherm was collected on Belsorp‐MAX (Bel Japan, Inc.). All samples were out gassed at 200 °C for 6 h under vacuum before measurement. The specific surface areas were calculated using the BET method. Specific surface area and the pore size distribution of samples were analyzed by Micromeritics ASAP‐2020 at 77 K. The measurements of XPS were conducted on a photoelectron spectrometer (PHI Quantera II ESCA System). The Mössbauer measurements were performed using a conventional spectrometer (Germany, Wissel MS‐500) in transmission geometry with constant acceleration mode. The velocity calibration was done with a 273 k α‐Fe absorber.

*Electrochemical Measurements*: Electrochemical measurements were performed on a CHI760E electrochemical workstation. The rotating disk electrode (diameter 5 mm) was used as working electrode. The graphite rod and Ag/AgCl electrode were used as auxiliary and reference electrode. All potentials reported are versus the reversible hydrogen electrode (RHE) in 1.0 m KOH with the scan rate of 5 mV s^−1^. All potential values versus the RHE were calibrated using two Pt electrodes in a hydrogen‐purged electrolyte, which calculated the RHE equation of *E*_(RHE)_ = *E*_(Ag/AgCl)_+0.989 V (Figure S16, Supporting Information). The different products ink was prepared by dispersing powers (10 mg) in the mixture solution of 1.5 mL water and 0.5 mL ethanol, then ultrasonically treated to form the inks.

*Electrode Modification*: 20 µL of solution containing catalyst was added on rotating disk electrode (RDE) with diameter of 5 mm and 3 µL Nafion (5%) ethanol solution was added when the electrode was dried at room temperature. Finally, the electrode was dried at 50 °C for 1 h. Voltage–time responses for the long‐term stability were monitored by chronopotentiometry measurements at a constant current density in 10 mA cm^−2^. All the electrochemical data were presented without IR compensation.

## Conflict of Interest

The authors declare no conflict of interest.

## Supporting information

Supplementary

Supplementary

Supplementary

Supplementary
