# Supplementary material for: Core–Shell Prussian Blue Analogs with Compositional Heterogeneity and Open Cages for Oxygen Evolution Reaction
Source: Adv Sci (Weinh). 2019 Feb 8;6(7):1801901. doi: 10.1002/advs.201801901 (PMC6446613; doi:10.1002/advs.201801901)
Supplement: Supplementary file 1 — Supplementary [file ADVS-6-1801901-s002.pdf]

Copyright WILEY-VCH Verlag GmbH & Co. KGaA, 69469 Weinheim, Germany, 2019.

ADVANCED SCIENCE
Open Access

Supporting Information

for *Adv. Sci.,* DOI: 10.1002/advs.201801901

Core–Shell Prussian Blue Analogs with Compositional Heterogeneity and Open Cages for Oxygen Evolution Reaction

Wuxiang Zhang, Hao Song, Yan Cheng, Chao Liu, Chaohai Wang, Muhammad Abdul Nasir Khan, Hao Zhang, Jizi Liu, Chengzhong Yu, Lianjun Wang, and Jiansheng Li**

Core-shell Prussian Blue Analogs with Compositional Heterogeneity and Open Cages for Oxygen Evolution Reaction

Wuxiang Zhang [a], Hao Song [b], Yan Cheng [c], Chao Liu [a, d], Chaohai Wang [a], Muhammad Abdul Nasir khan [a], Hao Zhang [a], Jizi Liu [e], Chengzhong Yu [b, d*], Lianjun Wang [a], Jiansheng Li [a*]

[a] Jiangsu Key Laboratory of Chemical Pollution Control and Resources Reuse, School of Environmental and Biological Engineering, Nanjing University of Science and Technology, Nanjing 210094, PR China

E-mail: lijsh@njust.edu.cn

[b] Australian Institute for Bioengineering and Nanotechnology, The University of Queensland, Brisbane, QLD 4072, Australia

E-mail: c.yu@uq.edu.au

[c] Department of Electronic Engineering, School of Information Science Technology, East China Normal University, Shanghai 200241, PR China

[d] School of Chemistry and Molecular Engineering, East China Normal University, Shanghai 200241, PR China

[e] Herbert Gleiter Institute of Nanoscience, Nanjing University of Science and Technology, Nanjing 210094, PR China

Figure S1. (a) ET characterizations of PBAs-5 in angles of 0°, ±30° and ±60°. The core-shell structure is clearly seen at 60 degree.

Figure S2. Tomographic analysis of Fe and Co elements spatial distribution.

Figure S3. (a) SEM and (b) TEM characterizations of PBAs-RT. (c) STEM images and (d) EDX line-scans of Fe and Co element obtained of PBAs-RT. EDX point analysis of Fe and Co element at PBAs-RT(e) and PBAs-5 (f).

Figure S4. FESEM and TEM characterizations of intermediate structures obtained at different time intervals: PBAs-1, PBAs-2, PBAs-3, PBAs-4 and PBAs-5.

Figure S5. (a) SEM, (b) TEM, (c) XRD and (d) EDX data of PBAs-6.

Figure S6. Hydrodynamic diameter of PBAs-1, PBAs-2, PBAs-3, PBAs-4 and PBAs-5.

Figure S7. The transformation of color from PBAs-1 to PBAs-5.

Figure S8. N_2 adsorption-desorption isotherms and pore size distributions of the PBAs1-5 samples.

Figure S9. EDX analysis of the powder PBAs-1, PBAs-2, PBAs-3, PBAs-4 and PBAs-5 samples.

Figure S10. High-resolution Co 2p (a-e) and Fe 2p (f-j) perform of the different stages of PBAs1-5.

Figure S11. Room temperature Mössbauer spectra of PBAs-5.

Figure 12, I. (a) SEM, (b) TEM and (c) HAADF images with EDX line-scans of Fe-Mn-PBAs-1, and (d) SEM, (e) TEM and (f) HAADF images with EDX line-scans of Fe-Mn-PBAs-2.

Figure 12, II. Fitting curve of the total Fe and Mn concentration in the supernatant solutions in different time (5 min to 12 h). Inset: the Mn/Fe ratio by EDX analysis of Fe-Mn-PBAs-1 and Fe-Mn-PBAs-2 in the solid samples.

Figure S13. (a) LSV curves of PB, commercial RuO_2, PBAs-5 and PBAs-6 (b) Tafel slopes of RuO_2 and PBAs-5 at a rotation rate of 1600 rpm.

Figure S14. EIS analysis with fitted curves of the OER catalysts.

Figure. S15. (a) SEM, (b) TEM image, (c) XRD patterns, (d) High-resolution Co 2p and (e) Fe 2p (f) and O1s perform of after-test PBA-5 catalysts.

Figure S16. RHE calibration in 1.0 M KOH solution.

Table S1. Synthetic strategy of bimetallic PBAs in recent years.

Table S2. The diameter and depth of the cavity with PBAs framework.

Table S3. N_2 adsorption-desorption parameters of PBAs1-5.

Table S4. Calculated chemical formula of PBAs-1 to PBAs-5.

Table S5. Room temperature Mössbauer parameters of PBAs-5.

Table S6. Comparison of OER activity for PBAs-5 and representative bimetallic based catalysts.

Table S7. The correlative parameters of EIS plots.

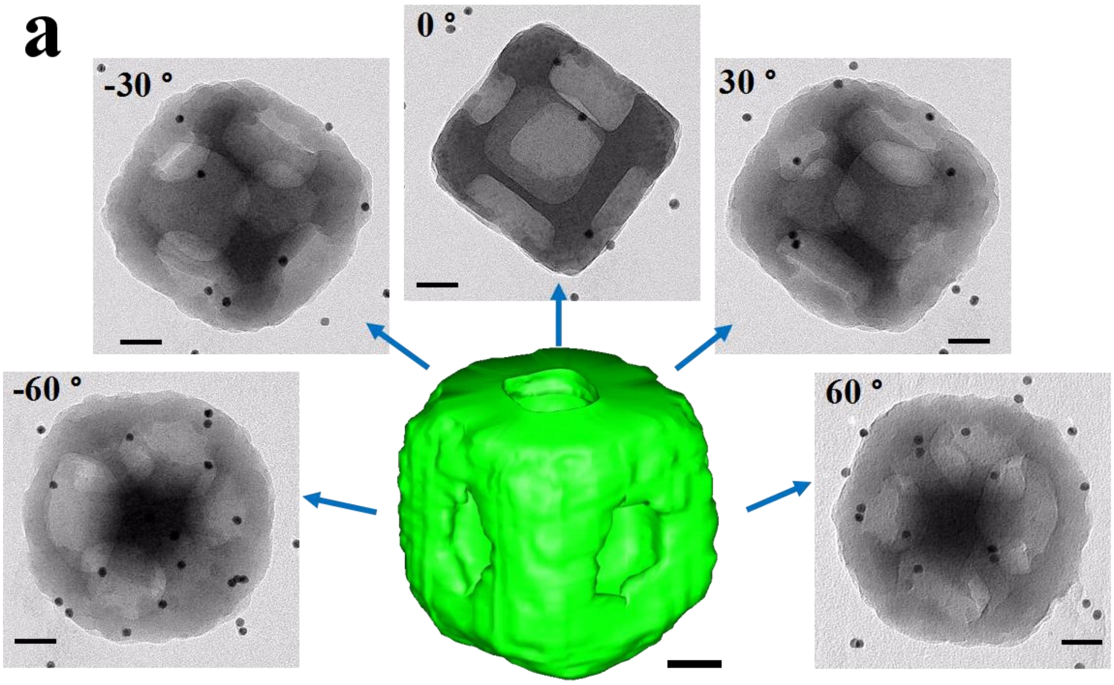

Figure S1. (a) ET characterizations of PBAs-5 in angles of 0°, ±30° and ±60°. The core-shell structure is clearly seen at 60 degree.

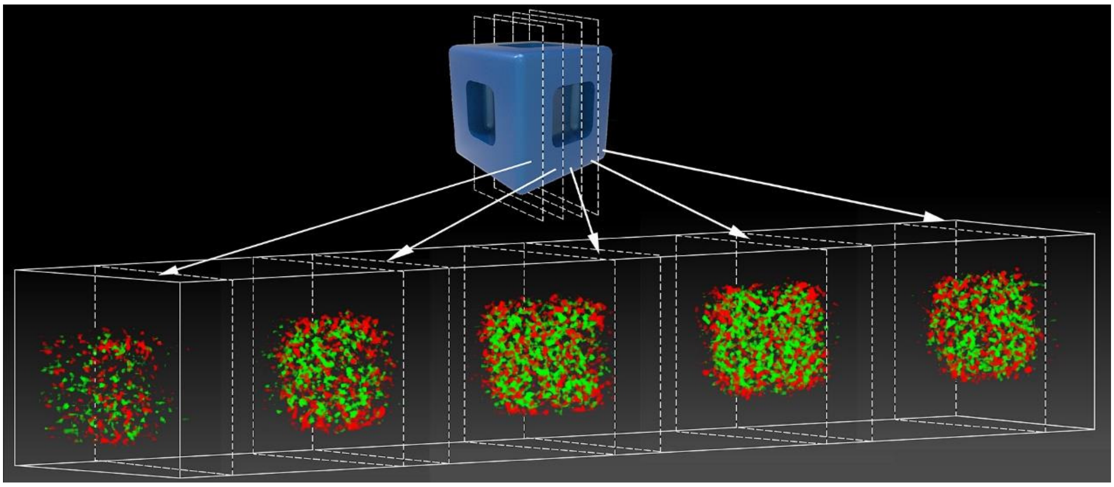

Figure S2. Tomographic analysis of Fe and Co elements spatial distribution.

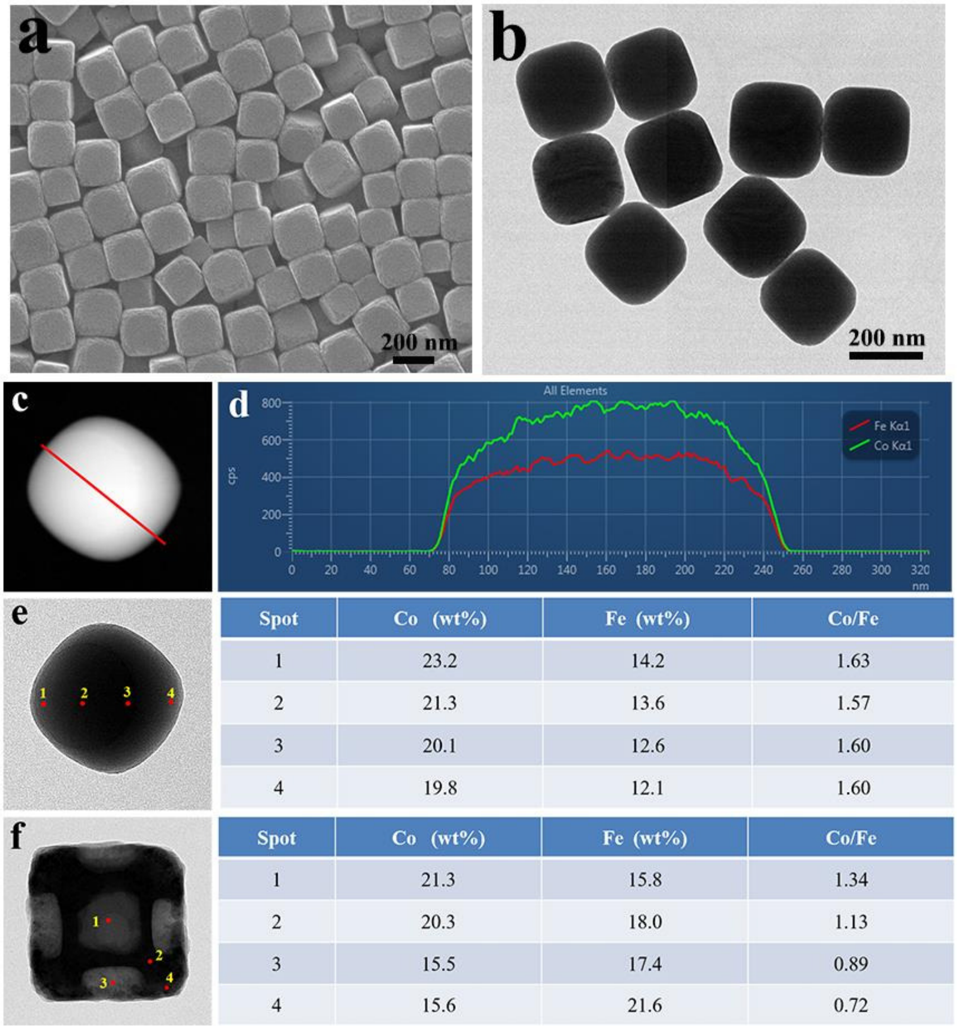

Spot	Co (wt%)	Fe (wt%)	Co/Fe
1	23.2	14.2	1.63
2	21.3	13.6	1.57
3	20.1	12.6	1.60
4	19.8	12.1	1.60

Spot	Co (wt%)	Fe (wt%)	Co/Fe
1	21.3	15.8	1.34
2	20.3	18.0	1.13
3	15.5	17.4	0.89
4	15.6	21.6	0.72

Figure S3. (a) SEM and (b) TEM characterizations of PBAs-RT. (c) STEM images and (d) EDX line-scans of Fe and Co element obtained of PBAs-RT. EDX point analysis of Fe and Co element at PBAs-RT(e) and PBAs-5 (f).

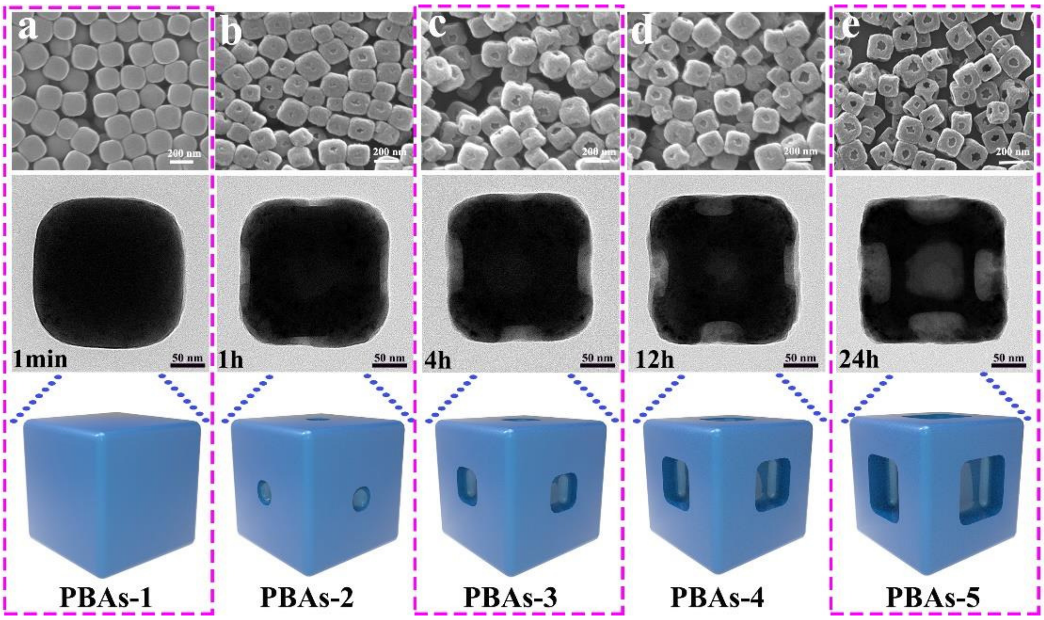

Figure S4. FESEM and TEM characterizations of intermediate structures obtained at different time intervals: PBAs-1, PBAs-2, PBAs-3, PBAs-4 and PBAs-5.

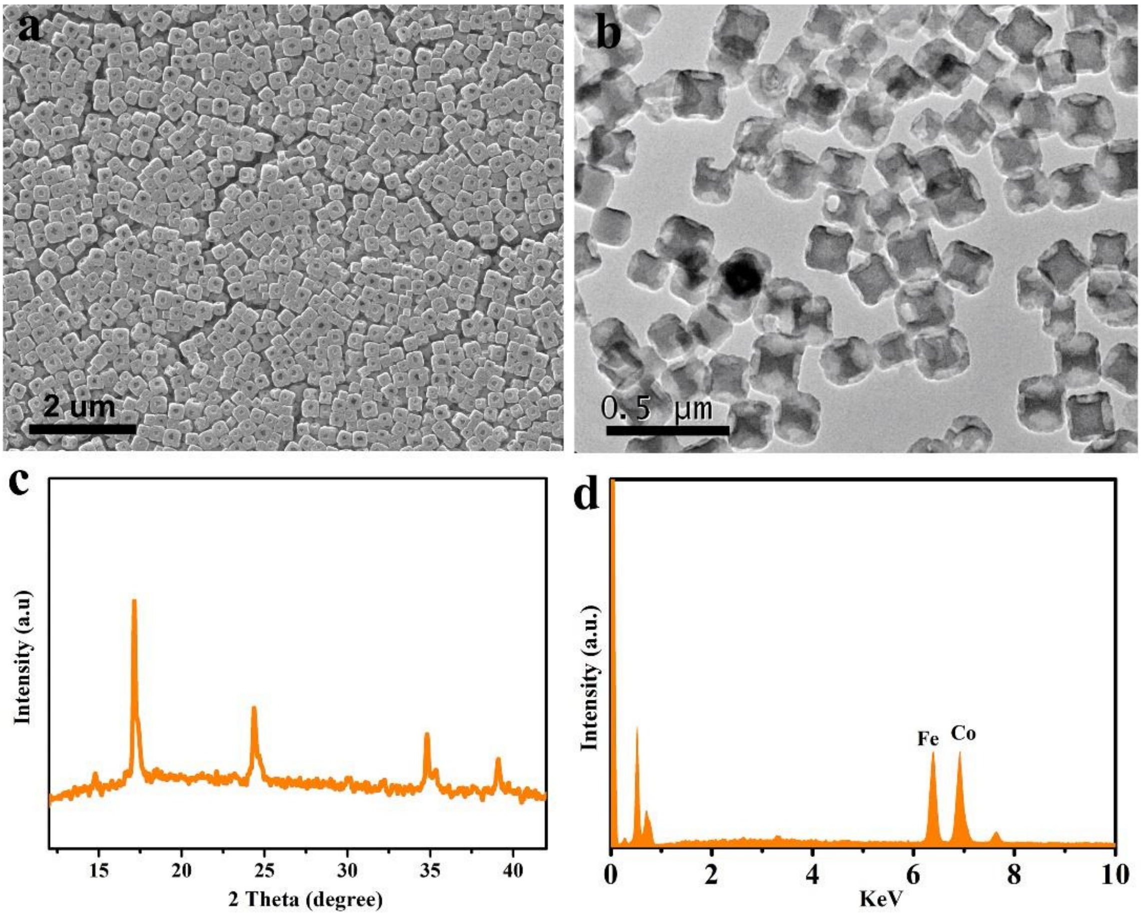

Figure S5. (a) SEM, (b) TEM, (c) XRD and (d) EDX data of PBAs-6.

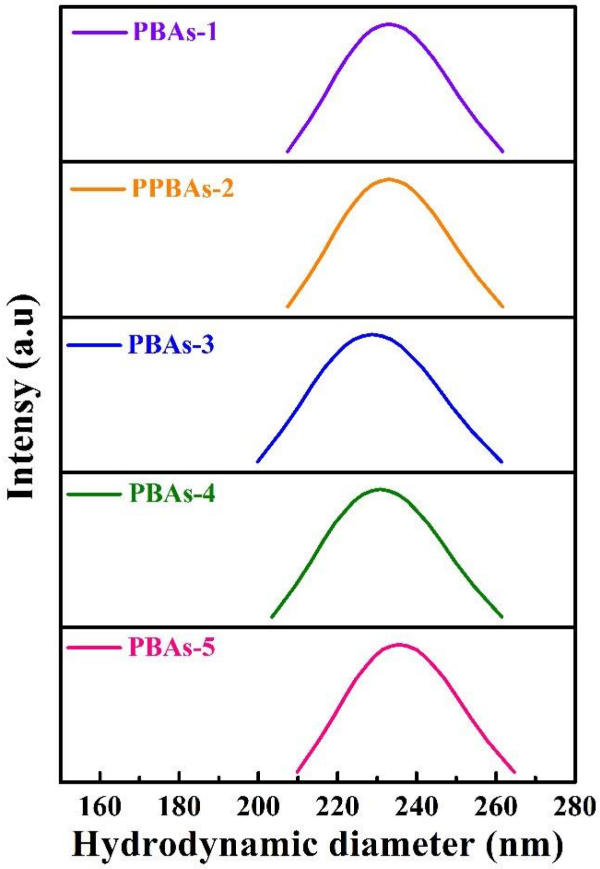

Figure S6. Hydrodynamic diameter of PBAs-1, PBAs-2, PBAs-3, PBAs-4 and PBAs-5.

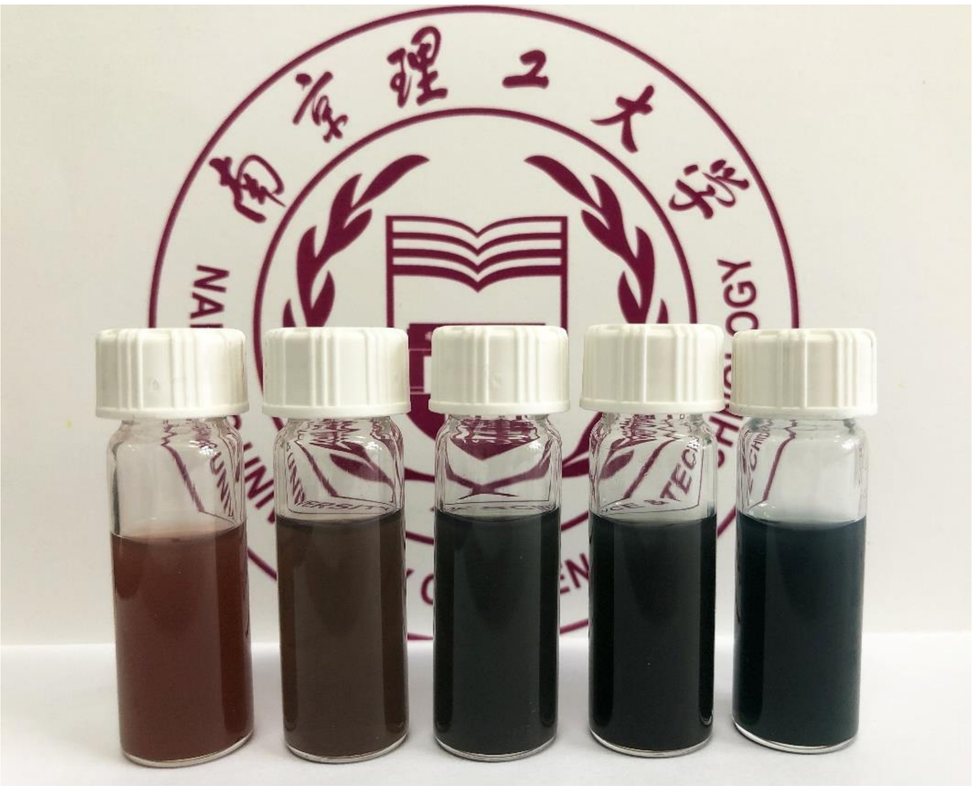

PBAs-1 PBAs-2 PBAs-3 PBAs-4 PBAs-5

Figure S7. The transformation of color from PBAs-1 to PBAs-5.

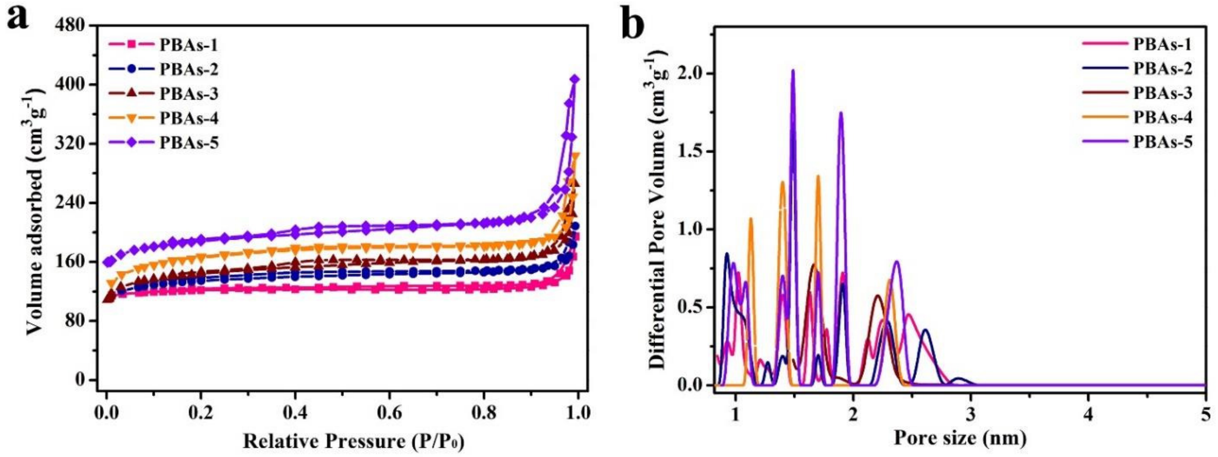

Figure S8. N_2 adsorption-desorption isotherms and pore size distributions of PBAs1-5 samples.

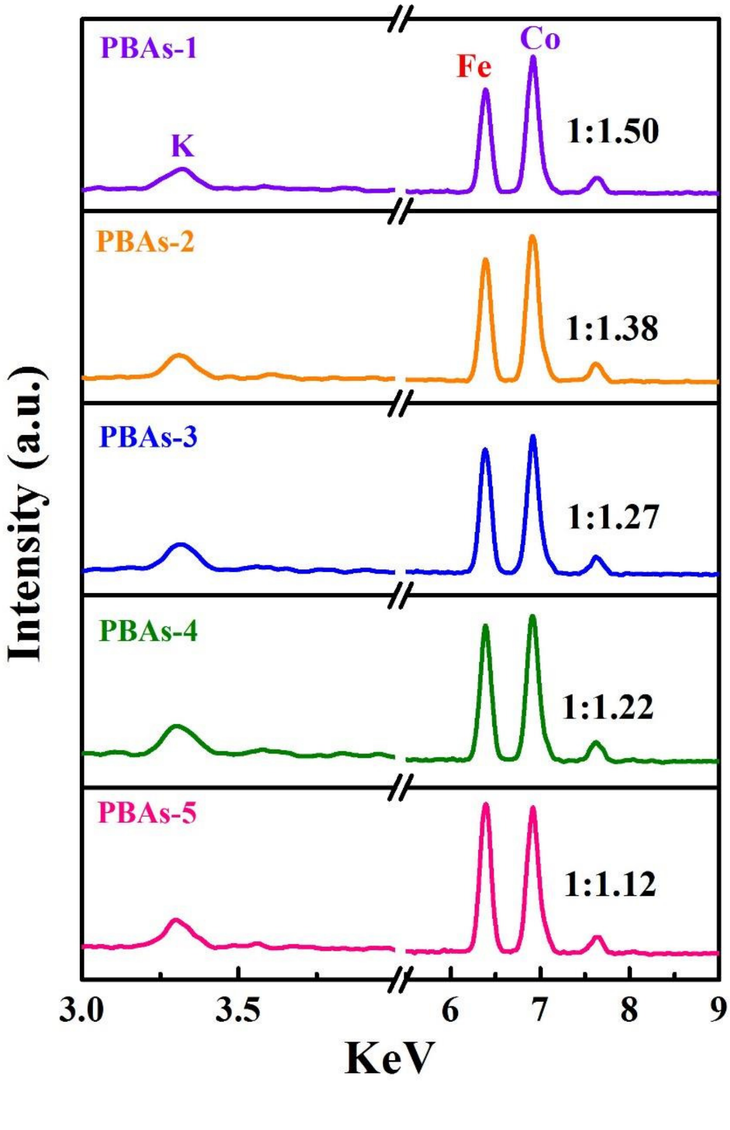

Figure S9. EDX analysis of the powder PBAs-1, PBAs-2, PBAs-3, PBAs-4 and PBAs-5 samples.

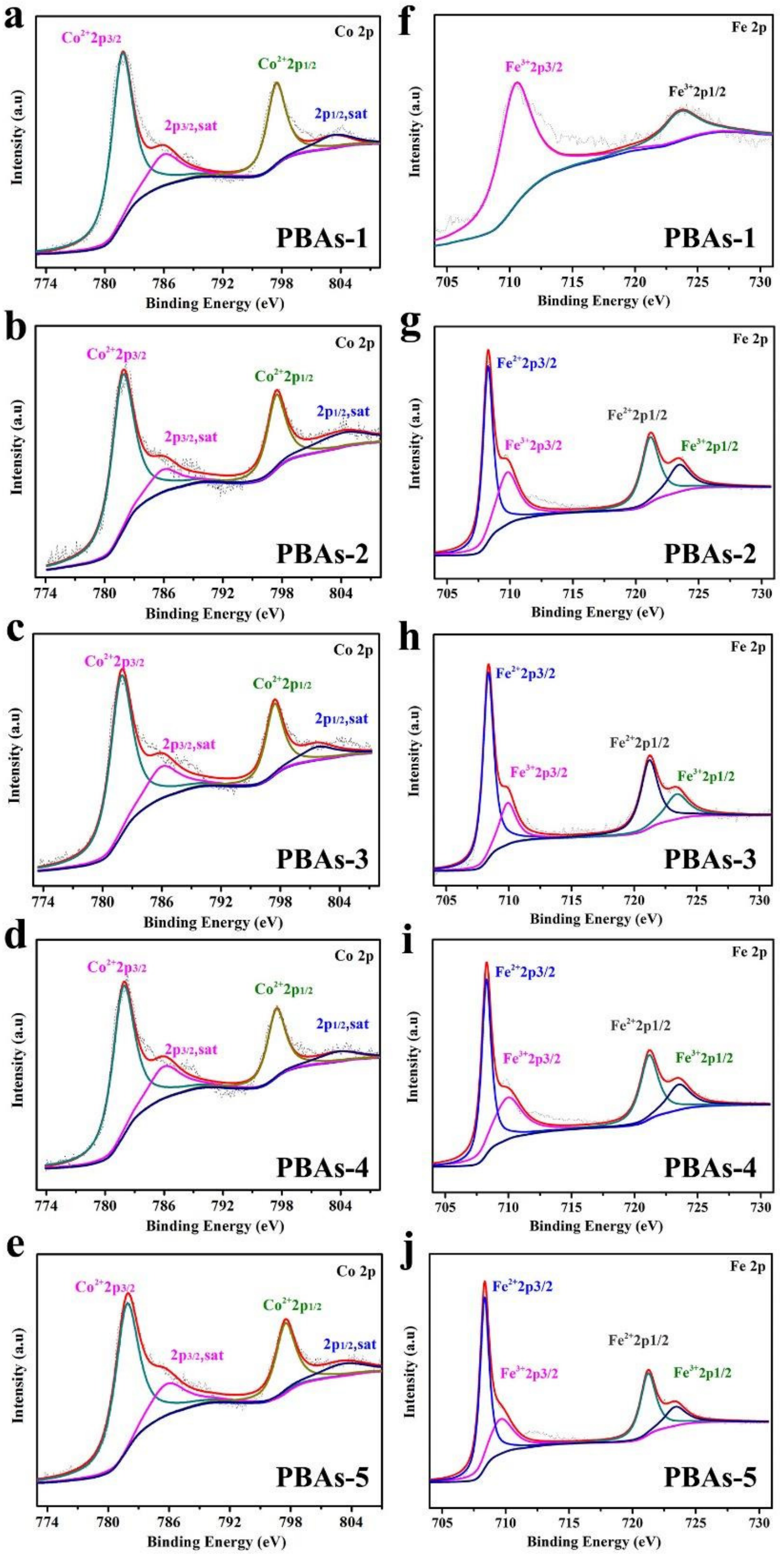

Figure S10. High-resolution Co 2p (a-e) and Fe 2p (f-j) perform of the different stages of PBAs1-5.

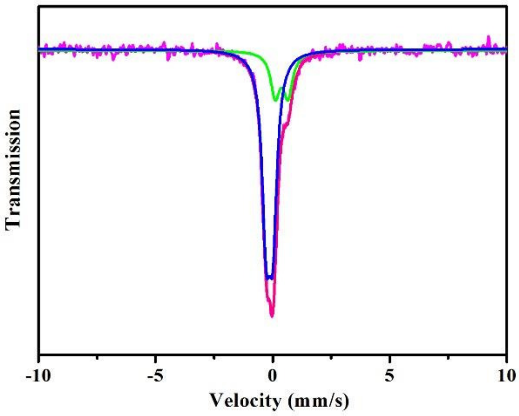

Figure S11. The RT Mössbauer spectra of PBAs-5. The green and blue solid lines represent the high-spin iron (III) and low-spin iron (II) spectral components, respectively.

The synthesis of Fe-Mn Prussian Blue analogs using the RCE strategy was also investigated. Unlike the Fe-Co PBAs system, no product was observed in Fe-Mn PBAs system at room temperature without hydrothermal treatment at 80 °C. This is due to the higher solubility product constant of $Mn^{II}_3[Fe^{III}(CN)_6]_2$ (Ksp=1.9 × 10^{-3}) than that of $Co_3[Fe^{III}(CN)_6]_2$ (Ksp=6.7×10^{-22}).[27] However, after hydrothermal treatment only for 5 min, Fe-Mn-PBAs-1 were formed due to the partial reduction of $[Fe(CN)_6]^{3-}$ by PVP and the even lower Ksp of Fe(II) based PBAs (Scheme 1). As shown in Figure S12, I, a, b, d, e, both Fe-Mn-PBAs-1 and Fe-Mn-PBAs-2 (hydrothermally treated for 12 h) exhibited a solid cubic morphology with a uniform size of ~220 nm, indicating that increasing the hydrothermal treatment time has negligible effect on the morphology of Fe-Mn-PBAs. However, compared to the Fe-Mn-PBAs-1, the ratio of Fe/Mn substantially increased as evidenced by EDX line-scanning images on Fe-Mn-PBAs-2 (Figure S12, I, c,f) and ICP-OES results (Figure S12, II) as well as EDX analysis (inset of Figure S12, II). Clearly, increasing the hydrothermal treatment time leads to continuous reduction and cation exchange (Fe^{2+} for Mn^{2+}). Nevertheless, only solid bimetallic Fe-Mn-PBAs were generated because there is no preformed $Mn^{II}_3[Fe^{III}(CN)_6]_2$ PBAs for selective etching in the RCE process.

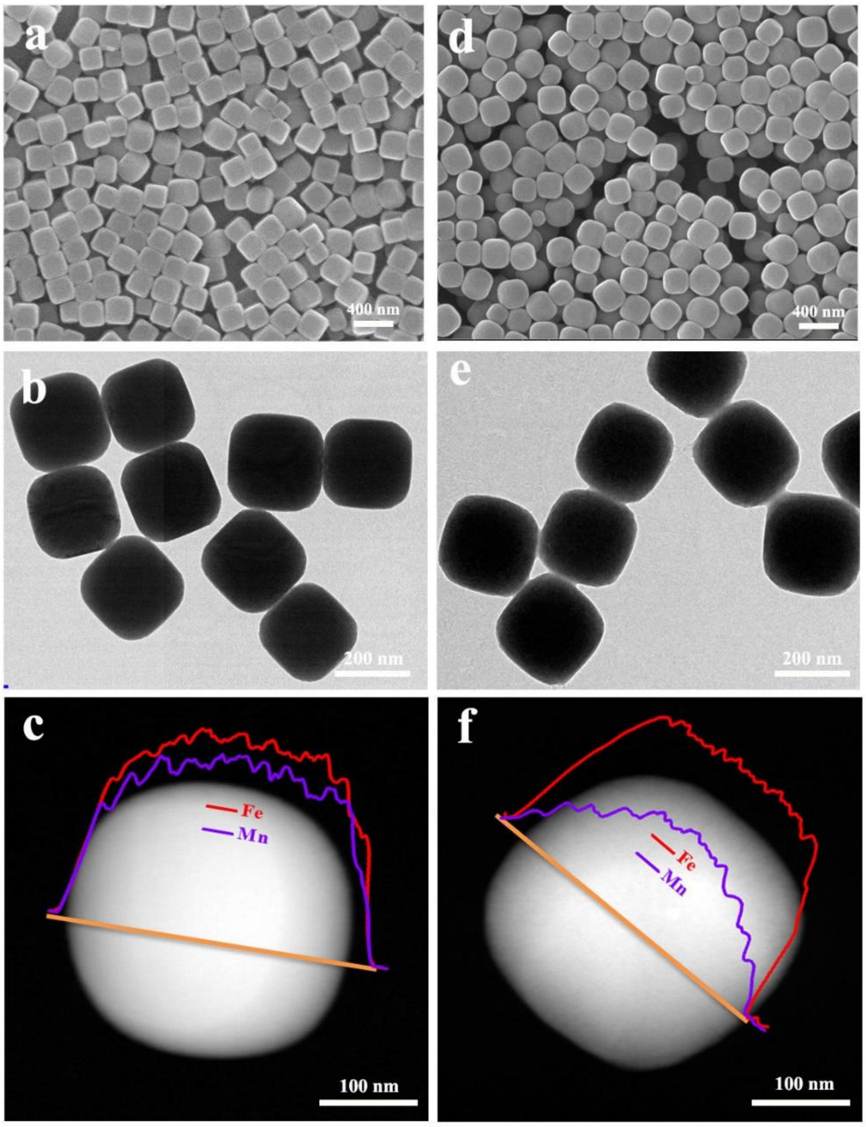

Figure S12, I. (a) SEM, (b) TEM and (c) HAADF images with EDX line-scans of Fe-Mn-PBAs-1, and (d) SEM, (e) TEM and (f) HAADF images with EDX line-scans of Fe-Mn-PBAs-2.

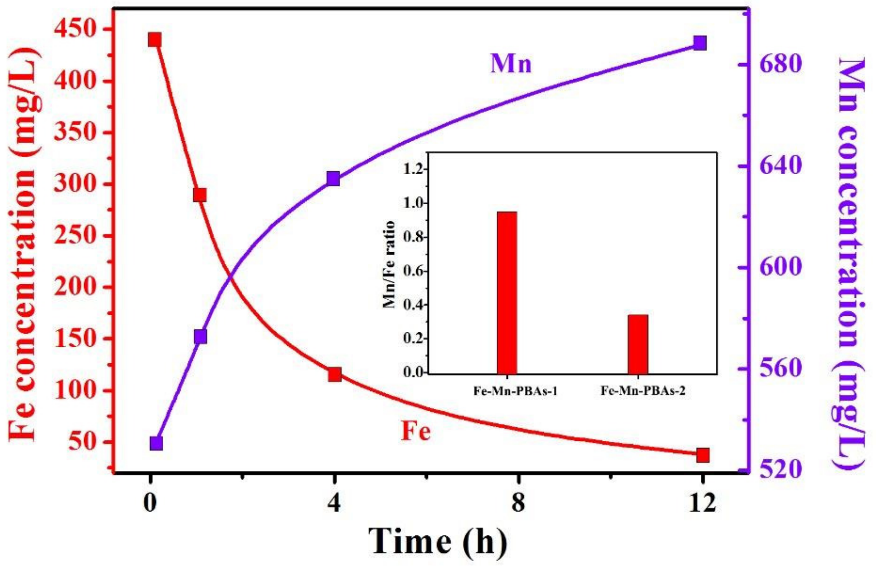

Figure S12, II. Fitting curve of the total Fe and Mn concentration in the supernatant solutions in different time (5 min to 12 h). Inset: the Mn/Fe ratio by EDX analysis of Fe-Mn-PBAs-1 and Fe-Mn-PBAs-2 in the solid samples.

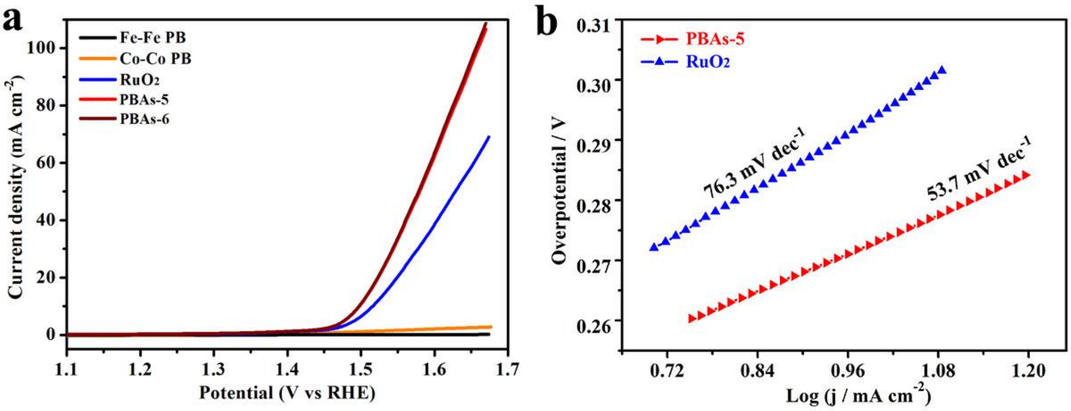

Figure S13. (a) LSV curves of solid pure Fe-Fe PB, Co-Co PB, RuO_2, PBAs-5 and PBAs-6 catalysts (b) Tafel slopes of RuO_2 and PBAs-5 at a rotation rate of 1600 rpm.

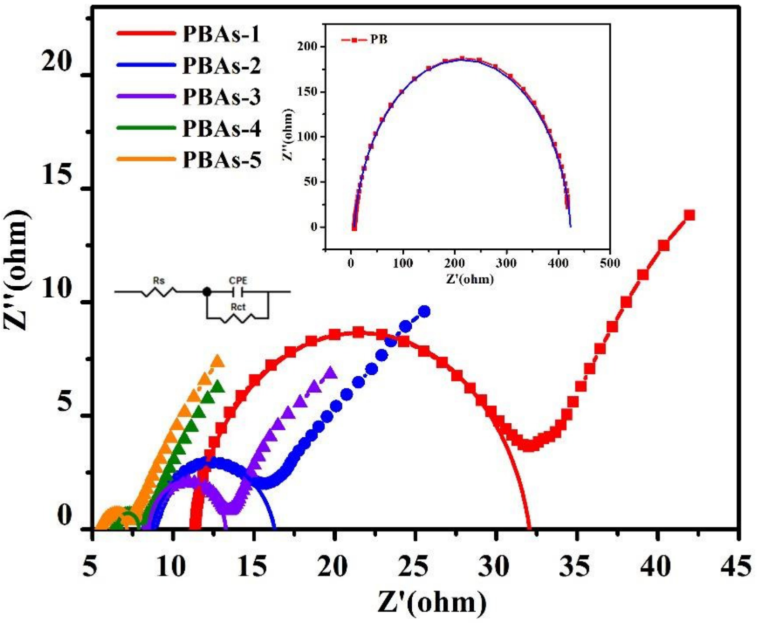

Figure S14. EIS analysis with fitted curves of the OER catalysts.

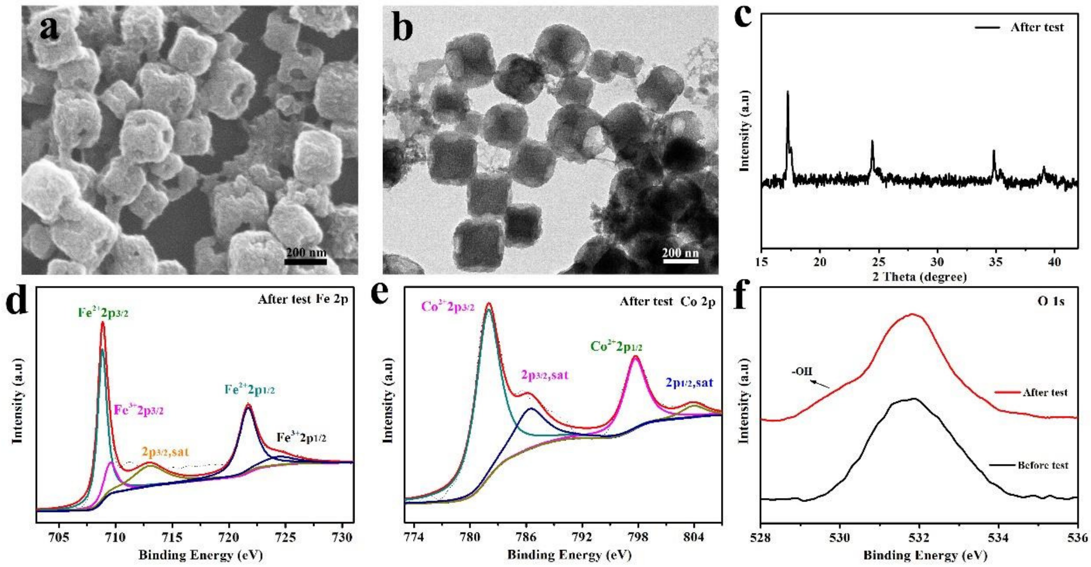

Figure. S15. (a) SEM, (b) TEM image, (c) XRD patterns, (d) High-resolution Fe 2p and (e) Co 2p (f) and O1s perform of after-test PBA-5 catalysts.

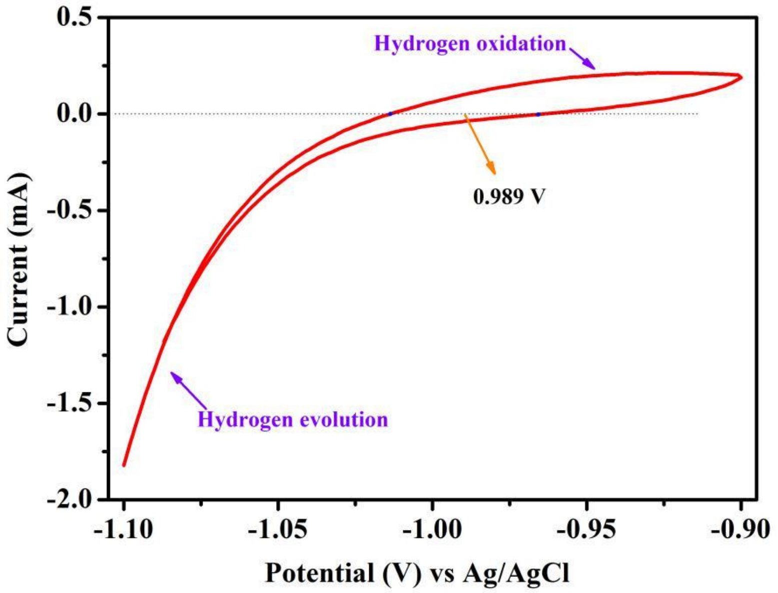

Figure S16. RHE calibration in 1.0 M KOH solution. $E_{(RHE)}=E_{(Ag/AgCl)}+0.989V$.

Table S1. Synthetic strategy of bimetallic PBAs in recent years.

**NO.**	**Method**	**Final products**			**Reference**
		**Structure**	**Chemical formula**	**Element composition**	
**1**	Cation exchange	Hollow submicroboxes	No mention	Homogeneous	1
**2**	Chemistry etching	Open nanoframe	$K_{0.16}Co[Fe(CN)_6]_{0.76}$, $K_{0.27}Co[Fe(CN)_6]_{0.88}$	Homogeneous	2
**3**	Chemistry etching	Nanocage	No mention	Homogeneous	3
**4**	Epitaxial growth	Frame-like/ cage-like	No mention	Homogeneous	4

	etching				
**5**	Epitaxial growth-etching	Hollow frame/box/cage	No mention	Homogeneous	5
**6**	Ion-modulation/ epitaxial growth	Core-shell<br>Hollow cage	$Mn^{II}_3[Fe^{III}(CN)_6]_2@Mn^{III}_2[Fe^{II}(CN)_4]_3$<br>$Mn^{III}_2[Fe^{II}(CN)_4]_3$	Heterogeneous<br>Homogeneous	6
**7**	**Reduction-cation exchange**	**Core-shell**	$\mathbf{Fe_xCo_{3-x}[Fe(CN)_6]_2}$	**Heterogeneous**	**This work**

[1] J. G. Wang, Z. Zhang, X. Zhang, X. Yin, X. Li, X. Liu, F. Kang, B. Wei, Nano Energy **2017,** 39, 647-653.

[2] G. Yilmaz, C. F. Tan, M. Hong, G. W. Ho, Advanced Functional Materials **2017,** 1704177.

[3] J. Nai, Y. Lu, L. Yu, X. Wang, X. W. Lou, Adv Mater **2017,** 29 (41).

[4] J. Nai, B. Y. Guan, L. Yu, X. W. Lou, Sci Adv **2017,** 3 (8), e1700732.

[5] J. Nai, J. Zhang, X. W. D. Lou, Chem **2018,** 4 (8), 1967-1982.

[6] S. Wu, G.Zhuang, J. Wei, Z. Zhuang, Y. Yu, J. M. Chem. A **2018,** 6 (37), 18234-18241.

Table S2. The diameter and depth of the cavity with PBAs framework.

**Sample**	**Cavity average diameter (nm)**	**Diameter range (nm)**	**Cavity average depth (nm)**	**Depth range (nm)**
PBAs-1	--	--	--	--
PBAs-2	35	25-48	15	12-18
PBAs-3	52	40-63	22	19-28
PBAs-4	60	46-79	31	27-36
PBAs-5	64	48-84	50	48-53

Table S3. N_2 adsorption-desorption parameters of PBAs1-5.

**Sample**	**BET surface area (m^2g^{-1})**	**Pore volume (m^2g^{-1})**	**Micropore volume (m^2g^{-1})**
PBAs-1	454.7	0.33	0.13
PBAs-2	461.9	0. 34	0.12
PBAs-3	468.6	0. 37	0.13
PBAs-4	484.7	0.39	0.13
PBAs-5	576.2	0.42	0.16

Table S4. Calculated chemical formula of PBAs-1 to PBAs-5.

**Sample**	**Chemical formula**
PBAs-1	$Co_3[Fe(CN)_6]_2$
PBAs-2	$Co_{2.95}Fe^{II}_{0.2}[Fe^{II}(CN)_6]_{0.75}[Fe^{III}(CN)_6]_{1.1}$
PBAs-3	$Co_{2.8}Fe^{II}_{0.36}[Fe^{II}(CN)_6]_{0.8}[Fe^{III}(CN)_6]_{1.04}$
PBAs-4	$Co_{2.76}Fe^{II}_{0.43}[Fe^{II}(CN)_6]_{0.95}[Fe^{III}(CN)_6]_{0.86}$
PBAs-5	$Co_{2.59}Fe^{II}_{0.61}[Fe^{II}(CN)_6]_{1.03}[Fe^{III}(CN)_6]_{0.76}$

Table S5. Room temperature Mössbauer parameters of PBAs-5.

Component	Assignment	IS (mm/s)	QS(mm/s)	Γ(mm/s)	Ratio
PBAs-5	HS Fe^{III}	0.386	0.549	0.239	0.32
	LS Fe^{II}	-0.1324	0.284	0.209	0.68

The IS is the isomer shift, QS is quadrupole splitting, Γ is the full line width at half maximum

Table S6. Comparison of OER activity for PBAs-5 and representative bimetallic based catalysts.

**Catalyst**	**Electrolyte**	**Current density (j, mAcm^{-2})**	**η_j (mV)**	**Tafel slope (mV dec^{-1})**	**Ref.**
(Fe-Co)PBAs-5	1 M KOH	10	271	53.7	This work
Fe−CoP/Ti	1 M KOH	100	310	67	Adv. Mater. **2017**, 29, 1602441.
Co-PBA-plasma-2h	1 M KOH	10	274	53	Adv. Energy Mater. **2018**, 1800085.
Ni–Co mixed oxide	1 M KOH	10	380	50	Adv. Mater. **2016**, 28, 4601.
Ni-Fe-OH@Ni_3S	1 M KOH	100	300	--	Adv Mater. **2017**, 29, 1700404.
Co-Fe-O frames	1 M KOH	10	290	62	Chem, **2018**, 4, 1.
NiFe LDH/NF	1 M KOH	10	240	NA	Science, **2014**, 345, 1593.

Table S7. The correlative parameters of EIS plots.

Sample	Rs (Ω)	Rct (Ω)
PB	17.4	413
PBAs-1	11.1	22.7
PBAs-2	8.7	9.6
PBAs-3	8.4	4.9
PBAs-4	6.3	2.8
PBAs-5	5.4	2.3
